# Drug-induced ciliogenesis in pancreatic cancer cells is facilitated by the secreted ATP-purinergic receptor signaling pathway

**DOI:** 10.18632/oncotarget.23335

**Published:** 2017-12-16

**Authors:** Niamat Ali Khan, Abhishek D. Garg, Patrizia Agostinis, Johannes V. Swinnen

**Affiliations:** ^1^ Laboratory of Lipid Metabolism and Cancer, Department of Oncology, LKI-Leuven Cancer Institute, KU Leuven-University of Leuven, Leuven, Belgium; ^2^ Cell Death Research and Therapy (CDRT) Lab, Department of Cellular and Molecular Medicine, KU Leuven-University of Leuven, Leuven, Belgium

**Keywords:** cilium, pannexin channels, pancreatic cancer, autocrine/paracrine signaling, extracellular adenosine triphosphate

## Abstract

Malignant transformation of cells is often accompanied by the loss of the primary cilium, a protruding microtubule-based sensory organelle, suggesting that it plays an “onco-suppressive” role. Therefore, restoration of the primary cilium is being explored as a new therapeutic approach to attenuate tumor growth. Recently, several commonly used chemotherapeutic drugs have been identified to induce the primary cilium in pancreatic cancer cells. The mechanisms by which these drugs re-express the cilium remain, however, enigmatic. Here, evaluation of a panel of diverse ciliogenic drugs on pancreatic cancer cell models revealed a significant positive relationship between drug-induced extracellular ATP, released through pannexin channels, and the extent of primary cilium induction. Moreover, cilium induction by these drugs was hampered in the presence of the ATP degrading enzyme, apyrase, and in the presence of the pan-purinergic receptor inhibitor, suramin. Our findings reveal that ciliogenic drug-induced re-expression of the primary cilium in pancreatic cancer cells is, at least in certain contexts, dependent on a hitherto unrecognized autocrine/paracrine loop involving the extracellular ATP-purinergic receptor signaling pathway that can be exploited in a therapeutic approach targeting at restoring the primary cilium.

## INTRODUCTION

Primary cilia are microtubule-based, antenna-like structures present on the surface of a wide variety of mammalian cells [1]. They serve as platforms for key signaling pathways that are critical for development and tissue homeostasis by specifically expressing crucial entities like ion channels, transporter proteins and receptors [2–4]. Hence, defects that compromise ciliary structure and/or function can have profound consequences for a cell [4]. Cilia dysfunction is considered to be a common event in many cancer types, including melanoma, breast cancer and pancreatic cancer [4]; and is associated with poor prognosis in patients [4–8]. Recently, a high-throughput screening based on the pancreatic CFPAC-1 cancer cell line delineated chemotherapeutic compounds with the ability to induce primary cilia in cancer cells, and paved the way to explore cilium induction as a hitherto unexploited concept in the context of cancer chemotherapy [9].

Armed with such novel ciliogenic drugs, we aimed to explore the mechanisms underlying cilium-induction in pancreatic cancer cells. Recent discoveries demonstrate that several anticancer modalities, including chemotherapeutic drugs, cause secretion of ATP from cancer cells [10–14]. Initially delineated in the context of neurotransmission, extracellular ATP now has emerged as an important messenger in a number of signaling paradigms [15, 16]. It facilitates cell-to-cell communication in both autocrine and paracrine manners in various pathological conditions [10, 15]. In the context of cancer, extracellular ATP has recently emerged as an important damage-associated molecular pattern (DAMP) [17], modulating both chemo-attraction and activation of innate immune cells in a paracrine manner [12, 18–20]. On the other hand, autocrine signaling elicited by extracellular ATP can also facilitate pro-cancerous processes e.g. increased proliferation and migration/invasion [21]. Thus, depending on the signaling context and target cells involved, extracellular ATP can exert both pro- and anti-cancerous effects [15, 16, 22]. Two mutually-exclusive pathways have been demonstrated to facilitate trafficking of extracellular ATP after treatment with anticancer therapy [17, 23] i.e. the classical secretory pathway (in case of photodynamic therapy) and the pannexin channels-based secretion (in case of chemotherapy and/or targeted therapy) [18, 24]. After release or secretion, extracellular ATP tends to signal through the P2 purinergic receptors on the surface of (target) cells [15].

Interestingly, two decades ago some studies had reported that exogenous addition of extracellular ATP can improve ciliary beat frequency (CBF) in epithelial cells derived from frog palate and esophagus [25, 26]. The modulation of CBF was further shown to occur through activation of purinergic receptors present on the cilium [3, 27, 28]. Consistent with these findings, mutant mice lacking purinergic receptors showed a significant reduction in CBF [29]. Collectively, these findings hinted towards a bi-directional link between primary cilia and extracellular ATP or purinergic receptor signaling, that had not been probed in the context of cancer cells, thus-far.

Following the above cues, we aimed to investigate to what extent and how cilia-inducing chemotherapeutic drugs (hereafter referred to as ciliogenic drugs) promote ciliogenesis in pancreatic cancer cells through the extracellular ATP-purinergic receptor signaling pathway. Here we show that in CFPAC-1 pancreatic ductal cancer cells that were used for the original screening for ciliogenic chemotherapeutics, several ciliogenic compounds exert their cilium-promoting effects at least in part through the extracellular ATP-purinergic receptor signaling pathway.

## RESULTS

### Ciliogenic chemotherapeutic drugs induce secretion of ATP in pancreatic cancer cells

**Figure 1:**
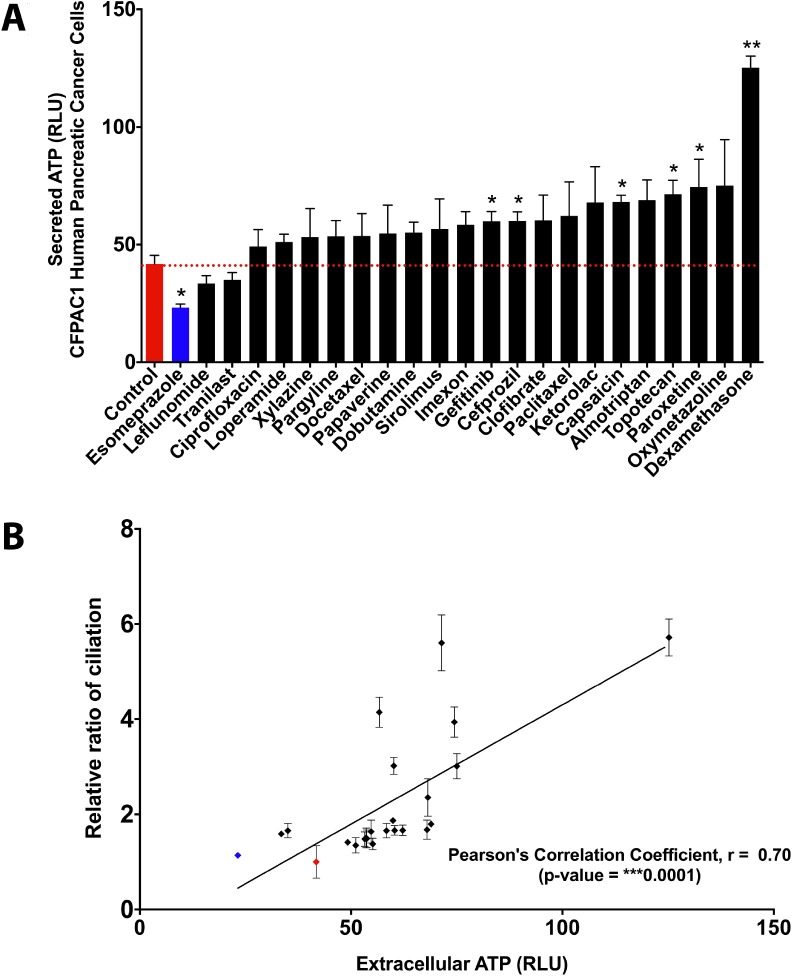
Ciliogenic chemotherapeutics tend to induce extracellular ATP in cancer cells Quantitative and correlative analysis of extracellular ATP upon exposure of CFPAC-1 cells to a panel of ciliogenic drugs (black bars and dots) and a non-ciliogenic drug (blue bar and dot) at 2 micromolar concentration for 96 hours. (**A**) The drugs are ranked in ascending order of their potency to release extracellular ATP into the culture medium as assessed by the measurement of bioluminescence based on luciferin-luciferase conversion principle. The red dotted line represents the basal level of extracellular ATP in the medium (represented as relative luminescence units or RLUs). (**B**) Regression analysis between extracellular ATP and cilia induction. Correlation was calculated by using Pearson correlation coefficient analysis. Data are presented as mean ± SEM, ^*^p ≤ 0.05, ^**^p ≤ 0.005, ^***^p ≤ 0.0005.

First of all, we wanted to assess whether extracellular ATP levels in fact correlate with re-expression of the primary cilium in cancer cells by ciliogenic chemotherapeutic drugs. To this end, we first investigated to what extent these drugs cause release/secretion of ATP in the extracellular medium [30]. To address this, human pancreatic cancer cells (CFPAC-1) were treated with a panel of 22 drugs that were previously shown to induce ciliogenesis in these cells [9]. The drug esomeprazole, which is incapable of inducing cilia [9], was included as negative control. Interestingly, 6 out of 22 drugs significantly increased the levels of extracellular ATP as compared to untreated control cells (Figure 1A). These results were reconfirmed in another pancreatic cancer cell line model PANC-1 for a selection of compounds (Supplementary Figure 1). While, most other ciliogenic compounds also induced a certain degree of ATP secretion/release, yet it was not statistically significant. Interestingly, a correlation analysis showed that drug-induced re-expression of primary cilia positively correlates (significantly) with increased secretion/release of ATP in these cancer cells (Figure 1B). These observations confirmed that certain potent ciliogenic drugs also tend to cause increased release/secretion of ATP.

### Exogenous ATP induces primary cilia in pancreatic cancer cells

**Figure 2:**
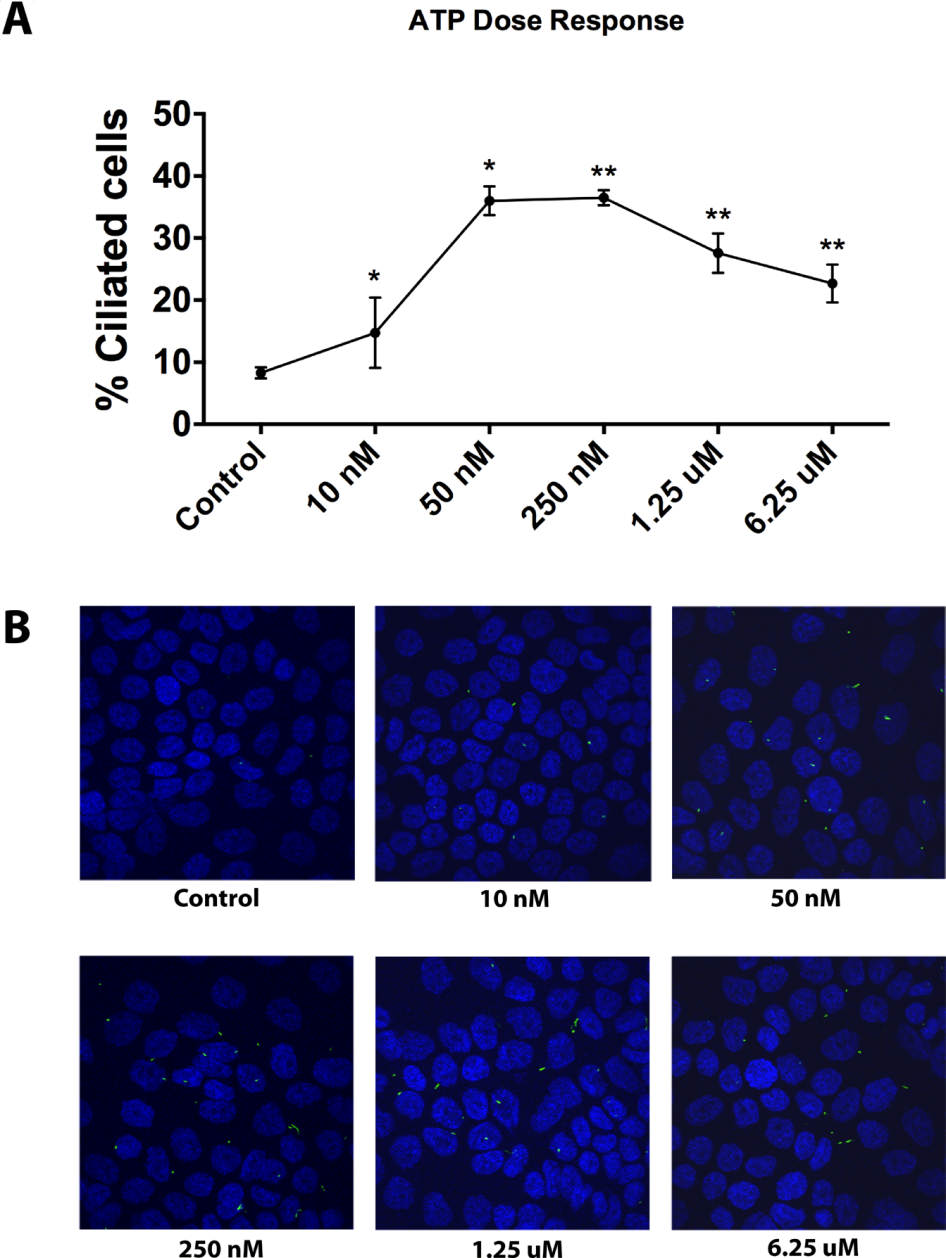
Effect of exogenous ATP on cilia induction in CFPAC-1 cells (**A**) Quantitative analysis of ciliogenesis upon treatment of cells with exogenous ATP at increasing concentrations, as assessed by confocal fluorescence microscopy. (**B**) Representative images of cells showing the effect of exogenous ATP on ciliation. Nuclei were stained with DAPI (blue) and cilia with an antibody against the cilium marker acetylated tubulin (green). All images were captured using Olympus Fluoview confocal microscope using a 40× objective lens. Data are presented as mean ± SEM, ^*^p ≤ 0.05, ^**^p ≤ 0.005, ^***^p ≤ 0.0005.

The above observations spurred us to assess whether exogenous ATP can in fact modulate ciliogenesis. Therefore, we exposed untreated CFPAC-1 cells to increasing concentrations of exogenously added ATP and visualized the primary cilium by confocal microscopy. A significant increase in the percentage of ciliated cells was observed already at nanomolar concentrations of exogenously added ATP. At higher micromolar concentrations the increase was less pronounced but still significant (Figure 2A and 2B). A similar effect was seen in PANC-1 cells (Supplementary Figure 2A and 2B). These results show that exogenous ATP enhances ciliogenesis in pancreatic cancer cells already at low concentrations that are in the range of the concentrations measured in the cultures after drug treatment (10–125 nM), suggesting a causative link between secreted ATP and cilia induction in pancreatic cancer cells.

### Degradation of drug-induced extracellular ATP suppresses ciliogenesis in pancreatic cancer cells

**Figure 3:**
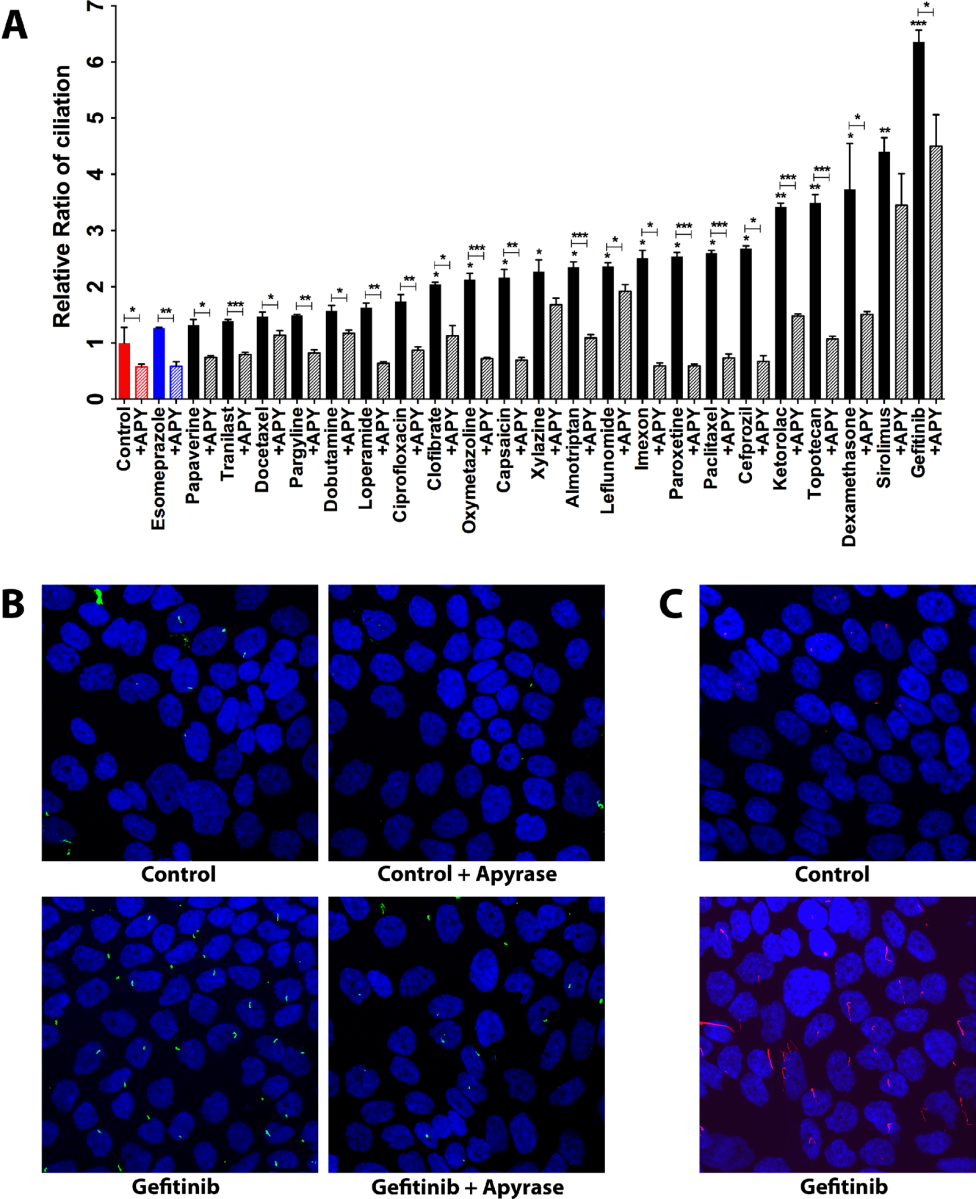
Effect of apyrase-mediated extracellular ATP degradation on ciliogenesis in CFPAC-1 cells exposed to ciliogenic drugs (**A**) Quantification of the effect of apyrase treatment on ciliogenesis. (**B**) Representative images showing the effect of apyrase on ciliogenesis of cells exposed to the indicated drugs. Nuclei were stained with DAPI (blue) and cilia with an antibody against the cilium marker acetylated tubulin (green). All images were captured using Nikon C2 Eclipse Ni-E confocal microscope using a 60× objective lens. Data are presented as mean ± SEM, ^*^p ≤ 0.05, ^**^p ≤ 0.005, ^***^p ≤ 0.0005. (**C**) Representative images showing the staining of cilia with an antibody against IFT88 (red), an alternative marker of the cilium.

To corroborate the link between secreted ATP and cilium induction, we assessed the ability of all the 22 ciliogenic compounds including the 6 ATP-releasing ones to modulate ciliogenesis in the presence of apyrase, a known ATP degrading enzyme. To this end, we applied an immunofluorescence microscopy-based phenotypic imaging strategy in a 96-well format using an IN Cell Analyzer, conceived by us previously [9]. In the presence of apyrase, the ability of ciliogenic drugs to increase the percentage of ciliated cells as well as the basal ciliogenesis was blunted, as compared to cancer cells treated in the absence of this ATP degrading enzyme (Figure 3A and Supplementary Figure 3A). These data were further substantiated by confocal microscopy for gefinitib, the most potent ciliogenic compound (Figure 3B and Supplementary Figure 3B). The induction of primary cilia visualized by acetylated tubulin staining was also substantiated by staining the cilia via IFT88, an alternative marker of the primary cilium (Figure 3C). These results provide further evidence that extracellular ATP is involved in cilium induction and thereby point towards the involvement of a secreted ATP-dependent autocrine mechanism in the re-expression of primary cilia in pancreatic cancer cells, especially by a subset of ciliogenic drugs that predominantly utilized this ATP-cilia axis.

Considering the above interesting observations, we wondered whether it is possible to recognize biological pathways or structure-function properties that are applicable to the 6 ciliogenic drugs that predominantly exploit the ATP-cilia axis versus the 18 other drugs that do not do so. To address this issue, we used the KEGG drug bioinformatics database to search for a biological pathway common to the 6 ciliogenic drugs that used increased ATP secretion to fuel cilia. Using this method we were unable to find a common biological pathway that could explain the ATP-based ciliogenesis of these 6 drugs. Next, we carried out a structure-function analyses using a cheminformatics approach based on the 2D chemical structures of the 22 drugs. 3D chemical structure-based cheminformatics was not employed since 3D structures were not available for all the above compounds thereby limiting the scope of that analysis. However, we failed to find any particular clustering pattern that can support a structure-function relationship that differentiates compounds facilitating cilia via heightened ATP secretion (Supplementary Figure 4). Overall, this implies that the structure of a particular compound cannot be reliably used to predict their ability to exploit the ATP-cilia axis.

### Pannexin channels mediate the secretion of extracellular ATP by ciliogenic drugs

**Figure 4:**
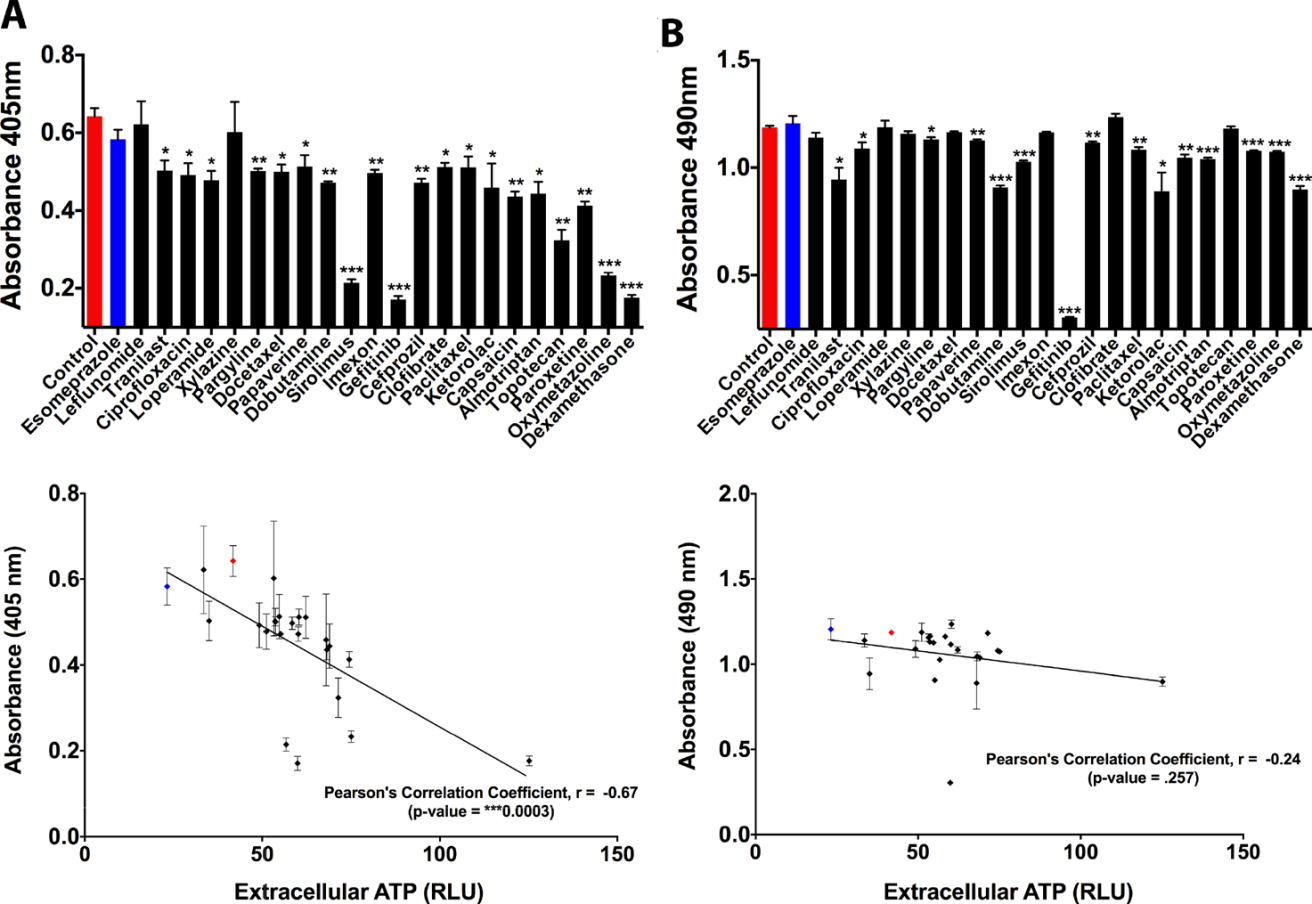
Effect of secreted ATP on proliferation and survival of CFPAC-1 pancreatic cancer cells (**A**) Quantitative analysis of cell proliferation upon secretion of ATP by exposure of cells to ciliogenic chemotherapeutic drugs (black bars and dots) and a non-ciliogenic drug (blue bar and dot). Changes in proliferation were measured by a BrdU incorporation assay. Lower panel shows correlative analysis between cell proliferation and secreted ATP. (**B**) Quantitative analysis of cell viability upon exposure of cells to ciliogenic chemotherapeutic drugs and a non-ciliogenic drug as measured by MTS assay. Lower panel displays the correlation between cell survival and secreted ATP in treated cells. Correlation was calculated by using Pearson correlation coefficient analysis. Data are presented as mean ± SEM, ^*^p ≤ 0.05, ^**^p ≤ 0.005, ^***^p ≤ 0.0005.

Beyond the above discussed effects which confirm that drug-induced ATP release affects ciliogenesis, chemotherapeutic drugs tend to also modulate the proliferation and survival of cancer cells – processes that have ability to modulate/exploit extracellular ATP. To this end, we deemed it crucial to analyze the overall correlation between secreted/released ATP and drug-induced effects on proliferation/survival. We observed an overall reduction in cellular proliferation following ciliogenic drug treatment, which interestingly correlated with an increase in the secretion/release of ATP (Figure 4A) thereby exposing a negative correlation between proliferation and extracellular ATP in this system. On the other hand, although most of the ciliogenic drugs compromised cellular survival to a certain extent, no statistically significant correlation was observed between cellular survival and ATP secretion (Figure 4B), suggesting the possibility of existence of an active ATP secretion pathway.

**Figure 5:**
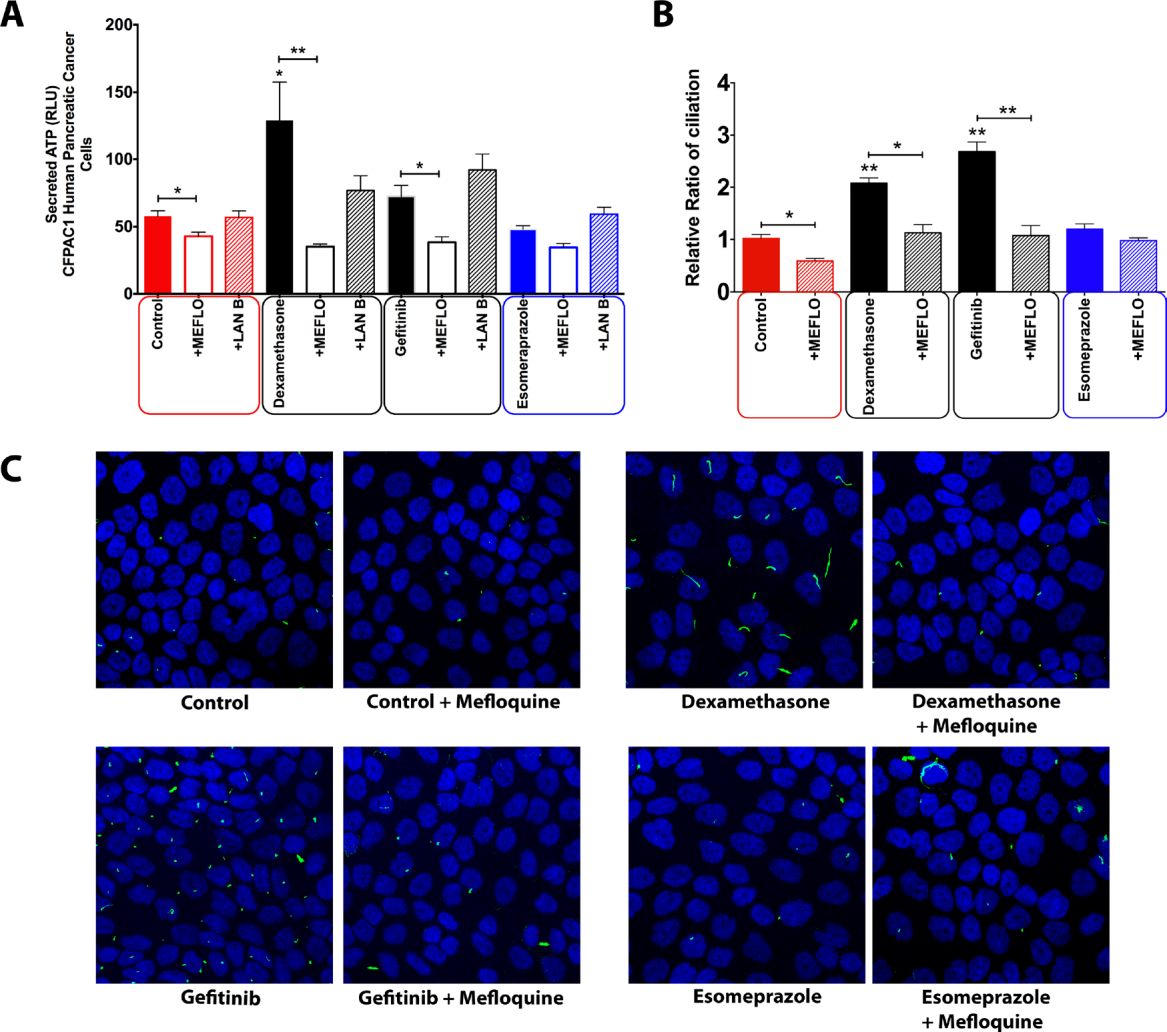
Effect of inhibition of different intracellular trafficking pathways on the concentration of extracellular ATP in CFPAC-1 cells treated with ciliogenic drugs (**A**) Quantification of extracellular ATP in cells treated with pannexin channel blocker (mefloquine), vesicular transport inhibitor (latrunculin B). (**B**) Quantification of the effect of mefloquine on ciliogenesis in CFPAC-1 cells. (**C**) Representative images showing the effect of pannexin channel blockade on ciliogenesis in CFPAC-1 cells exposed to a selection of ciliogenic drugs. Nuclei were stained with DAPI (blue) and cilia with an antibody against the cilium marker acetylated tubulin (green). All images were captured using Nikon C2 Eclipse Ni-E confocal microscope using a 60× objective lens. Data are presented as mean ± SEM, ^*^p ≤ 0.05, ^**^p ≤ 0.005, ^***^p ≤ 0.0005.

Although the cellular survival assay suggested that at least some extracellular ATP in our set-up might be passively released, the lack of a statistically significant correlation between survival and ATP release (Figure 4B) prompted us to explore whether ATP is secreted by the studied pancreatic cancer cells in an active manner; and to what extent this is crucial for ciliogenesis. To investigate the involvement of the two main ATP secretion mechanisms [17] we treated cells with well-established small molecule inhibitors each targeting different players in putative ATP secretion pathways. Vesicular transport mediated by actin was inhibited by the actin polymerization inhibitor latrunculin B; and pannexin-based release was inhibited by the pannexin channel inhibitor mefloquine. Latrunculin B pre-treatment failed to reduce drug-induced ATP secretion (Figure 5A), thereby ruling out a major role of vesicular and/or secretory anterograde transport in ATP secretion in this set-up. However, when pannexin channels were blocked with mefloquine, a significant reduction in the extracellular ATP was observed, indicating the involvement of this mechanism in ATP secretion induced by certain drugs (Figure 5A). The role of pannexins-based ATP secretion in our set-up was further corroborated by the observation that CFPAC-1 cells pre-treated with mefloquine and subsequently exposed to ciliogenic drugs showed a significant reduction in the percentage of ciliated cells (Figure 5B and 5C).

### P2 purinergic receptors are involved in drug-induced ciliogenesis

**Figure 6:**
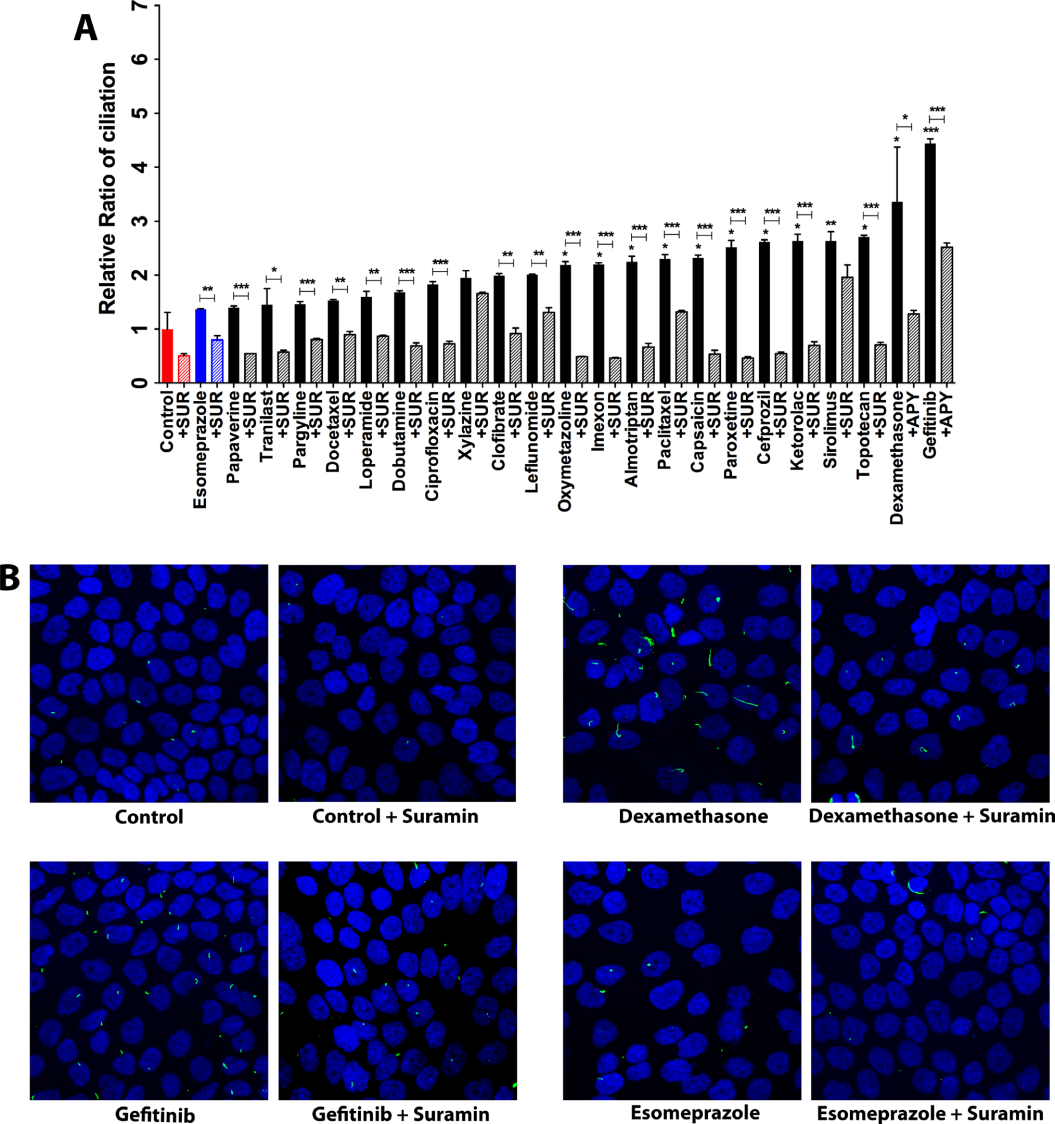
P2 purinergic receptor blockade reduces ciliogenesis (**A**) Effect of suramin, a blocker of P2 purinergic receptors on ciliogenesis in CFPAC-1 cells exposed to ciliogenic drugs. (**B**) Representative images of CFPAC-1 cells showing the effect of suramin on ciliation in untreated and treated cells. Nuclei were stained with DAPI (blue) and cilia with an antibody against the cilium marker acetylated tubulin (green). All images were captured using Nikon C2 Eclipse Ni-E confocal microscope using a 60× objective lens. Data are presented as mean ± SEM, ^*^p ≤ 0.05, ^**^p ≤ 0.005, ^***^p ≤ 0.0005.

**Figure 7:**
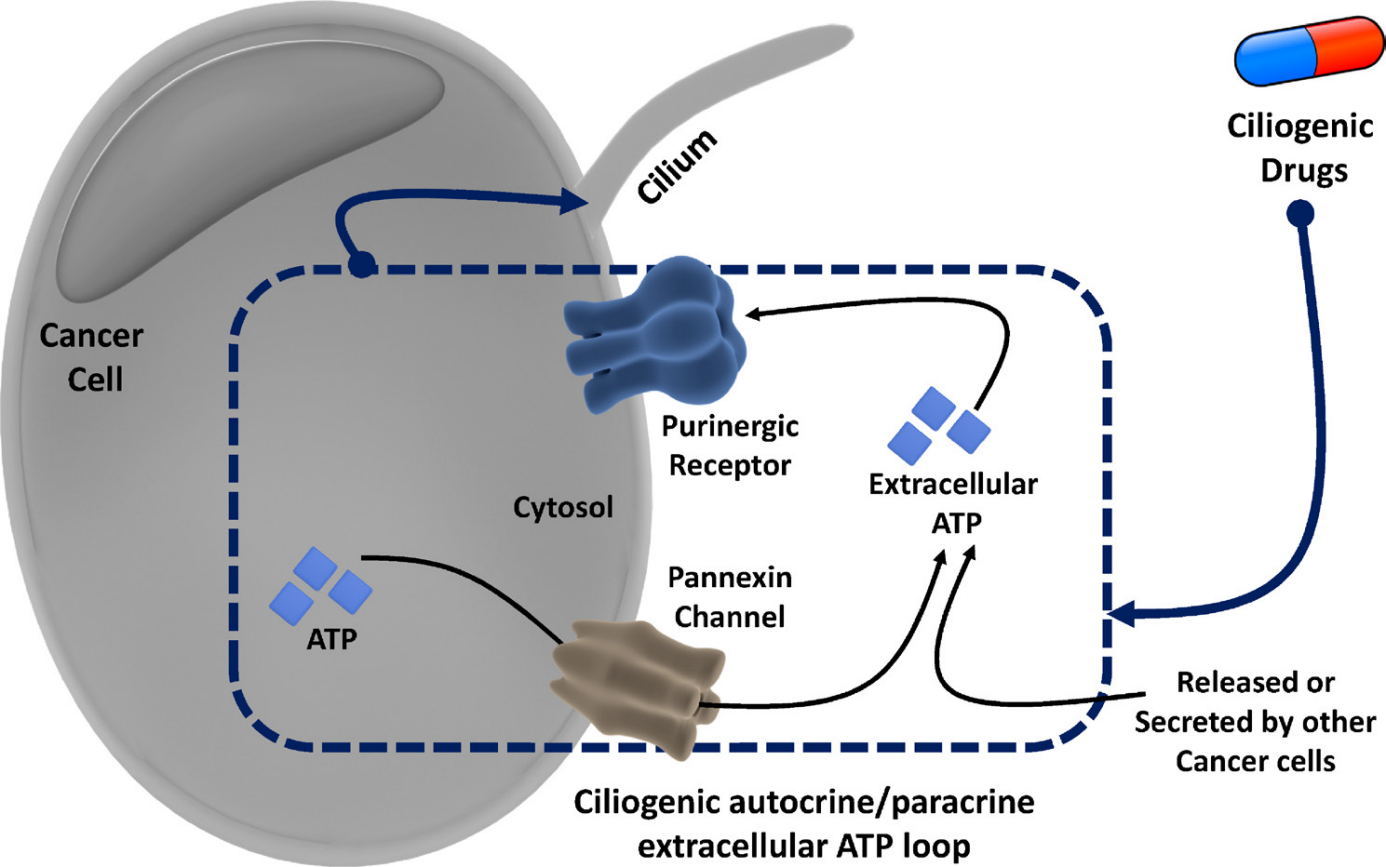
Overview of the proposed autocrine/paracrine loop model of extracellular ATP-mediated ciliogenesis by ciliogenic chemotherapeutics through activation of the pannexin pathway and purinergic signaling

As mentioned previously, extracellular ATP tends to largely signal through the P2 purinergic receptors. Since the increased extracellular ATP had a positive effect on ciliogenesis, we decided to probe the involvement of the extracellular ATP-P2 purinergic receptor link in the induction of primary cilia in CFPAC-1 cells by ciliogenic drugs. To this end, we exposed cells treated with ciliogenic drugs to suramin, a pan-P2 receptor inhibitor, and examined to what extent this interferes with ciliogenesis. Indeed, a significant suppression of ciliogenesis was observed in cells treated with the drugs when suramin was present (Figure 6A and 6B). Thus, our data show that both degrading ATP (thus removing the ligand) or inhibiting its main receptor exert the same effect, suggesting that the extracellular ATP-P2 purinergic receptor signaling arc is involved in the induction of primary cilia in pancreatic cancer cells (Figure 7).

## DISCUSSION

In a recent study we had shown that various commonly used chemotherapeutic drugs restore ciliogenesis in cancer cells [9]. Since the cilium uses the same structural elements as the centrosome that is required for cell division and proliferation, ciliogenesis is believed to put a brake on the proliferation of cells, including many cancer cells. This is also the reason behind cilium induction being regarded as a novel therapeutic mechanism [9]. Here, we demonstrate that certain drugs promote ciliogenesis, at least in part, through a hitherto unrecognized autocrine/paracrine loop involving the extracellular ATP-purinergic receptor-signaling pathway (Figure 7). We found that a subset of ciliogenic compounds stimulate ATP release, which positively correlates with their ciliogenic ability. Consistent with previous studies [18, 24], using chemical inhibitors targeting different potential players in ATP-secretion pathways we found that specific ciliogenic drugs stimulate the active secretion of ATP mainly through pannexin channels. This active ATP secretion pathway might co-exist with some passive release of ATP [30], at least in the case of certain ciliogenic drugs that induce cell death in a fraction of the cancer cells. Extracellular ATP in turn promotes ciliogenesis as revealed by the treatment of cells with varying concentrations of exogenous ATP, by blocking P2 purinergic receptors, which are known to mediate many effects of extracellular ATP [15] and by co-treatment with the ATP-degrading enzyme apyrase [30]. Thus, a picture emerges in which the extracellular ATP may act in both an autocrine manner (ATP secreted by a live cell that acts on the same cell) and a paracrine manner (ATP released from dead/dying cells or secreted by live cells that acts on other live cells) to induce ciliogenesis in pancreatic cancer cells (Figure 7).

This involvement of the extracellular ATP-purinergic receptor pathway in the cilium-inducing effect of certain chemotherapeutic drugs is of particular interest in view of the emerging central role of purinergic signaling in many cellular events including inflammation and wound healing [31–33]. Our observation of a statistically significant negative correlation between extracellular ATP levels and overall proliferation of pancreatic cancer cells is particularly intriguing in view of the divergent effects of extracellular ATP on cell proliferation [15, 34–36] and suggest that the mitigating effects of extracellular ATP-induced ciliogenesis on proliferation may at least partially account for this discrepancy. This notion needs proper molecular analysis and warrants further examination in a tumor context *in vivo*, to what extent this newly delineated function of extracellular ATP-purinergic signaling contributes to the anticancer effects of the studied drugs in pancreatic and other cancers.

While we have provided evidence for the role of autocrine/paracrine signaling by the extracellular ATP in drug-induced ciliogenesis; it should be considered that this pathway may not be the only existing mechanism. The action of chemotherapeutic drugs tends to be pleiotropic, consisting of both on-target as well as off-target therapeutic effects. In view of the complexity of cilia-inducing signaling it is likely that other signaling pathways also play a major role. Moreover, considering that extracellular ATP also has a crucial role in the modulation of inflammation [17, 37–40], and as a large number of anti-inflammatory drugs have been reported to induce primary cilia in cancer cells [9], it would be interesting to investigate the relationship between ciliogenesis and inflammation and the role of extracellular ATP in this context. Further elucidation of these and other pathways will be of interest to fully exploit the cilium-inducing property of common chemotherapeutics in a more clinical setting.

## MATERIALS AND METHODS

### Reagents and drugs

Esomeprazole, Pargyline, Leflunomide, Clofibrate, Ciprofloxacin, Ketorolac, Paroxetine, Dobutamine, Tranilast, Loperamide, Xylazine, Capsaicin, Papaverine, Oxymetazoline, Dexamethasone, Docetaxel, Topotecan, Paclitaxel were purchased from Sigma. Almotriptan Malate and Sirolimus were purchased from Selleckchem, Gefitinib from Invivogen, Cefprozil Monohydrate from Abcam and Imexon from MicroSource Discovery Systems Inc.; ATP, Apyrase, Suramin and Mefloquine were purchased from Sigma; Latrunculin B was purchased from Abcam.

### Cell culture and drug treatments

Both the pancreatic cancer cells CFPAC-1 and PANC-1 were obtained from ATCC. CFPAC-1 cells were cultured in RPMI-1640 medium (Life Technologies) whereas PANC-1 cells were maintained in DMEM medium (Life Technologies). Both media were supplemented with 10% FBS (Life Technologies) and incubated at 37°C in 5% CO_2_. For drug treatments, cells were seeded in 96-well plates at a density of 10,000 cells per well containing 200 µl of culture medium supplemented with 10% FBS and incubated in a humidified incubator at 37°C and 5% CO2 for 48 hours. This was followed by refreshment of the medium with culture medium containing 2% FBS. Assays were performed by treating the cells with drugs as indicated: for induction of primary cilia, the cells were treated with ciliogenic drugs. Hydrolysis of ATP was achieved by treating cells with Apyrase. Purinergic receptor signaling in cells was blocked by treatment with Suramin. Disruption of the secretory pathway was carried out by Latrunculin B treatment. Mefloquine was used to block the pannexin pathway.

### Immunofluorescence and automated imaging for primary cilia

To stain primary cilia, chemotherapeutics-treated and untreated cells were chemically fixed with 4% Formaldehyde (Merck), permeabilized with 0.1% Triton X100 (Merck) in DPBS, blocked with 1% BSA (Applichem) in DPBS, and incubated with 1:1000 dilution of anti-acetylated tubulin antibody (Sigma) or 1:500 dilution of anti-IFT88 antibody (Cat. No. 13967-1-AP, Proteintech) for 1 hour followed by incubation with 1:1000 dilution of a fluorescent secondary antibody (Life Technologies, AlexaFluor 488 and 594) for 1 hour. Nuclei were stained with Hoechst-33258 (Calbiochem). Images were acquired using an IN Cell Analyzer (GE Healthcare) at 20× magnification and were analyzed using IN Cell Developer software. In order to minimize the signal-to-noise ratio, 20 randomized fields per well were imaged at a single plane of focus.

### Confocal microscopy

CFPAC-1 and PANC-1 cells were seeded on glass coverslips in 12-well plates containing 1 ml culture medium per well and allowed to grow to 30% confluency. This was followed by replacement of culture medium with medium containing 2% FBS and treatment of cells with drugs as previously indicated. To visualize the primary cilium, treated and untreated cells were fixed with 4% formaldehyde, permeabilized with 0.1% Triton X100 in DPBS, blocked with 1% BSA in DPBS, incubated with 1:1000 dilution of anti-acetylated tubulin antibody for 1 hour, followed by incubation with 1:1000 dilution of fluorescent secondary antibody for 1 hour. The coverslips were mounted on glass slides and stained for nuclei with DAPI (Vector Laboratories, Vectashield). Images of primary cilia were captured by acquiring Z-stacks using Olympus FluoView-FV 1000 or Nikon C2 Eclipse Ni-E confocal laser scanning microscope using 60X oil immersion lens.

### Measurement of extracellular ATP

Extracellular ATP was measured using an ATP Bioluminescent assay kit (Sigma) based on the luciferin-luciferase conversion principle, according to the manufacturer’s instructions, as described previously [30]. Bioluminescence was measured by optical top reading using a FlexStation 3 microplate reader (Molecular Devices Inc., Sunnyvale, CA, USA).

### Proliferation assay

Proliferation assay was performed by measuring BrdU incorporation using 5-Bromo-2-deoxy-uridine Labeling and Detection Kit III (Cat No. 11444611001) in accordance with the manufacturer’s protocol.

### Cell viability assay

The viability of cells in drug treated cultures was determined by measuring the bioreduction of MTS tetrazolium compound using the CellTiter96 AQueous One Solution Cell Proliferation Assay kit (Promega). The assay was performed by following the protocol provided with the kit.

### Statistical analysis and cheminformatics analysis

Statistical analysis of data was performed using GraphPad Prism version 6 for Mac OS X (GraphPad Software, San Diego, California, USA). All data are expressed as mean ± SEM. Significance level of Student’s *t*-test was set at *p* < 0.05. Cheminformatics analysis was performed using the Structure Clustering utility of the NCBI database (accessed from this link: https://pubchem.ncbi.nlm.nih.gov/assay/assay.cgi?p=clustering). This methodology utilized Single Linkage algorithm harnessing the Tanimoto score calculated from the 2D structure fingerprint. Further bioinformatics analysis was performed using the KEGG drug database (http://www.genome.jp/kegg/drug/).

## SUPPLEMENTARY MATERIALS FIGURES



## Abbreviations

ATP: adenosine triphosphate
DAMPs: damage-associated molecular patterns
