# Supplementary material for: Drug-induced ciliogenesis in pancreatic cancer cells is facilitated by the secreted ATP-purinergic receptor signaling pathway
Source: Oncotarget. 2017 Dec 16;9(3):3507–18. doi: 10.18632/oncotarget.23335 (PMC5790479; doi:10.18632/oncotarget.23335)
Supplement: Supplementary file 1 [file oncotarget-09-3507-s001.pdf]

Drug-induced ciliogenesis in pancreatic cancer cells is facilitated by the secreted ATP-purinergic receptor signaling pathway

SUPPLEMENTARY MATERIALS

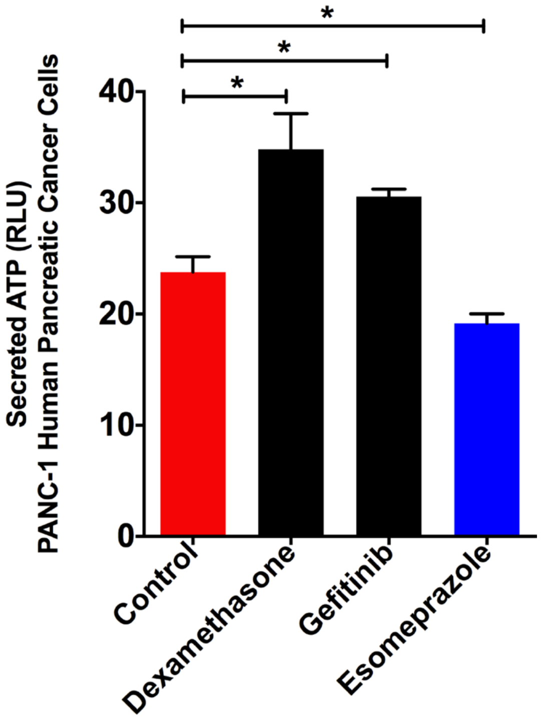

Supplementary Figure 1: Ciliogenic chemotherapeutics induce extracellular ATP in pancreatic cancer cell line PANC-1. Quantitative analysis of ATP secretion upon exposure of PANC-1 cells to a selection of ciliogenic drugs (black bars) and a non-ciliogenic drug (blue bar) at 2 micromolar concentration for 96 hours (**A**) as assessed by the measurement of bioluminescence based on luciferin-luciferase conversion principle. Data are presented as mean ± SEM, $^{*}p \leq 0.05$, $^{**}p \leq 0.005$, $^{***}p \leq 0.0005$.

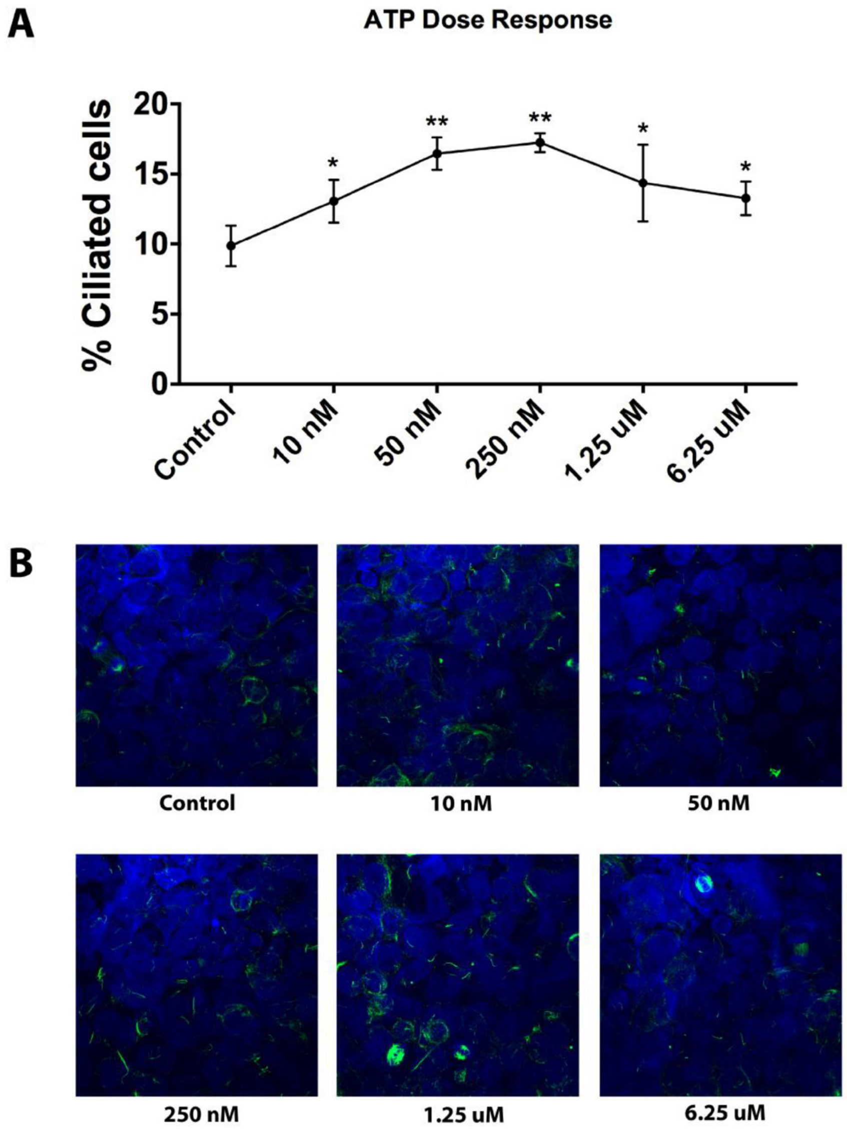

Supplementary Figure 2: Effect of exogenous ATP on cilia induction in PANC-1 cells. (**A**) Quantitative analysis of ciliogenesis in cells treated with increasing concentrations of exogenous ATP, as assessed by confocal fluorescence microscopy. (**B**) Representative images showing the effect of exogenous ATP on ciliation in PANC-1 cells. Nuclei were stained with DAPI (blue) and cilia with an antibody against the cilium marker acetylated tubulin (green). All images were captured using Olympus Fluoview confocal microscope using a 40× objective lens. Data are presented as mean ± SEM, $^{*}p \leq 0.05$, $^{**}p \leq 0.005$, $^{***}p \leq 0.0005$.

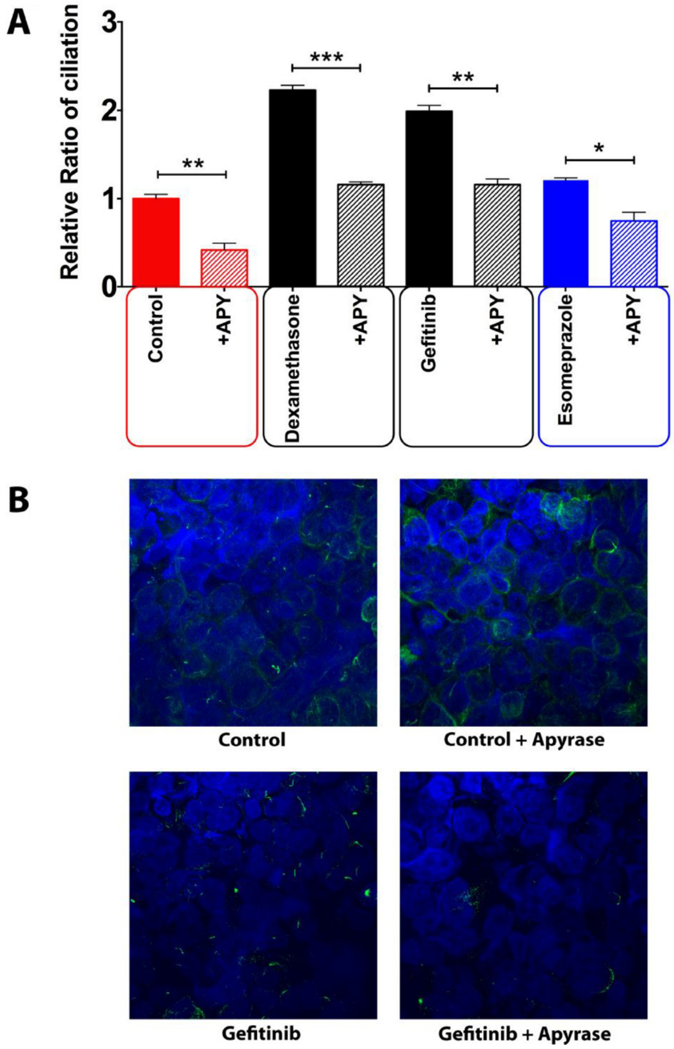

Supplementary Figure 3: Apyrase-mediated degradation of extracelluar ATP in pancreatic cancer cells PANC-1 exposed to ciliogenic drugs. (**A**) Quantitative analysis of the effect of apyrase treatment on ciliogenesis. (**B**) Representative images showing the effect of apyrase on ciliogenesis in cells treated with indicated drugs. Nuclei were stained with DAPI (blue) and cilia with an antibody against the cilium marker acetylated tubulin (green). Data are presented as mean ± SEM, $^{*}p \leq 0.05$, $^{**}p \leq 0.005$, $^{***}p \leq 0.0005$.

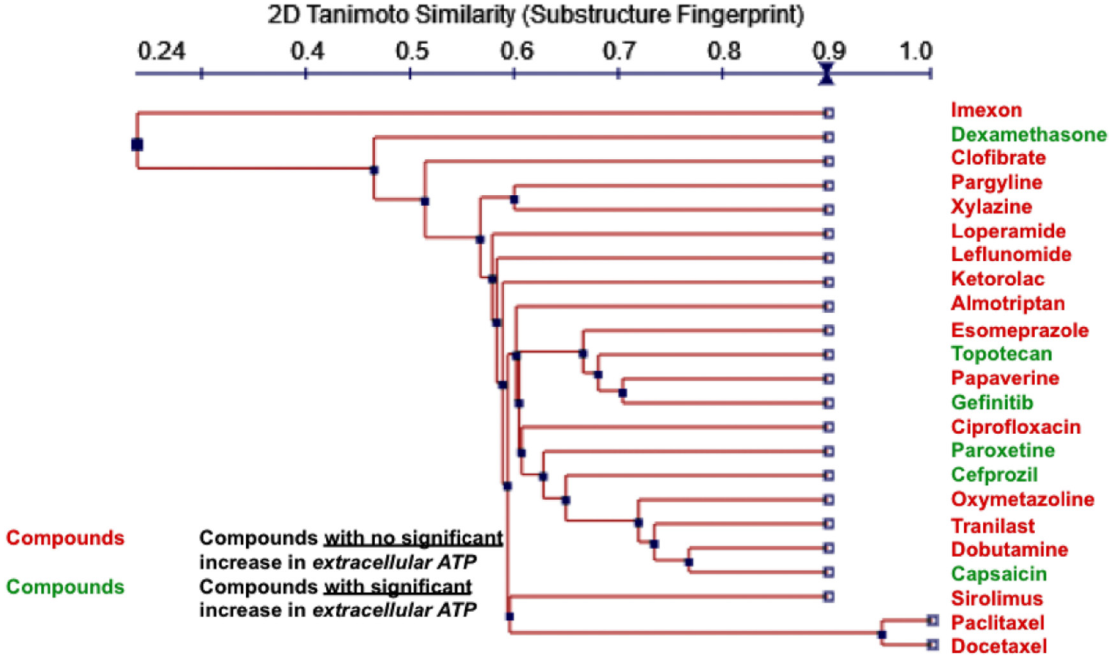

Supplementary Figure 4: Cheminformatics analysis of the structure-function relationship between 22 drugs and their ability to induce ciliogenesis and ATP secretion.
